# Supplementary material for: High nitrogen inhibits biomass and saponins accumulation in a medicinal plant Panax notoginseng
Source: PeerJ. 2023 Feb 21;11:e14933. doi: 10.7717/peerj.14933 (PMC9951802; doi:10.7717/peerj.14933)

# SUPPLEMENTARY FIGURE

**Figure S1** Diurnal variation of photosynthetic active radiation (PAR) for 3 days.


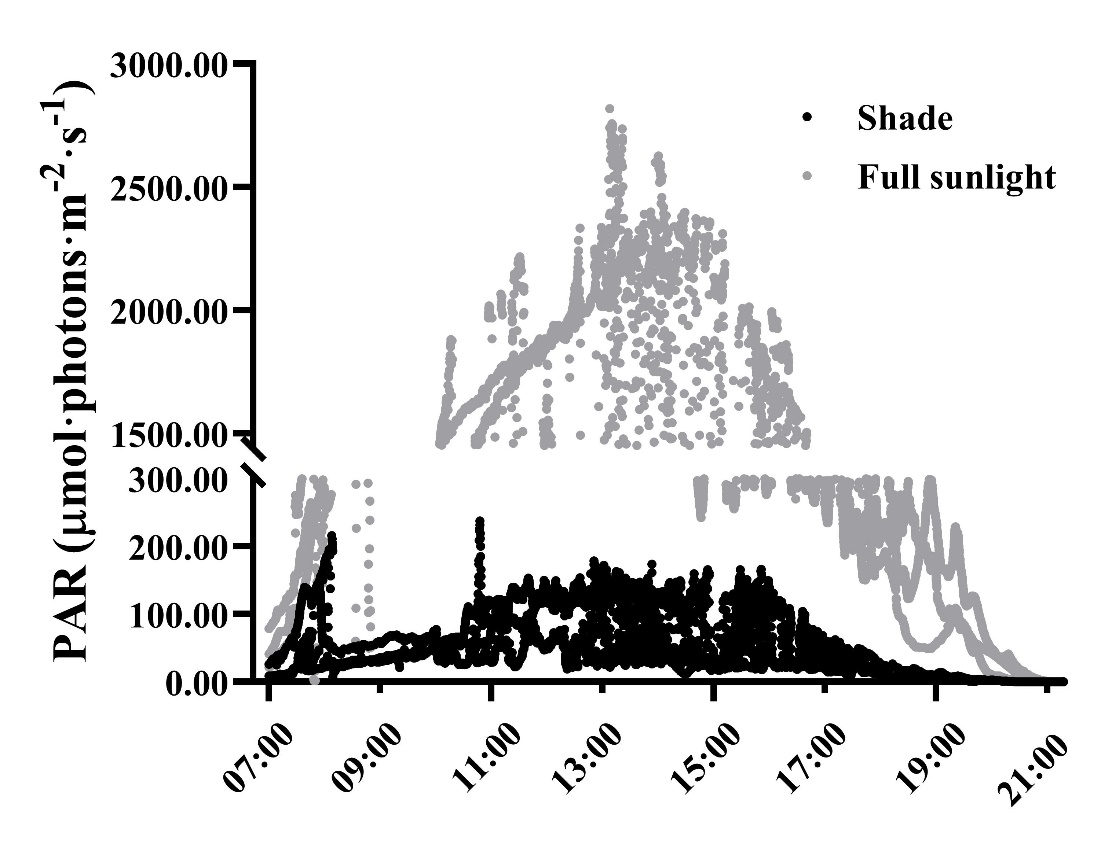


**Figure S2** Leaf phenotypic traits of three-year-old *Panax notoginseng* grown under different nitrogen levels.


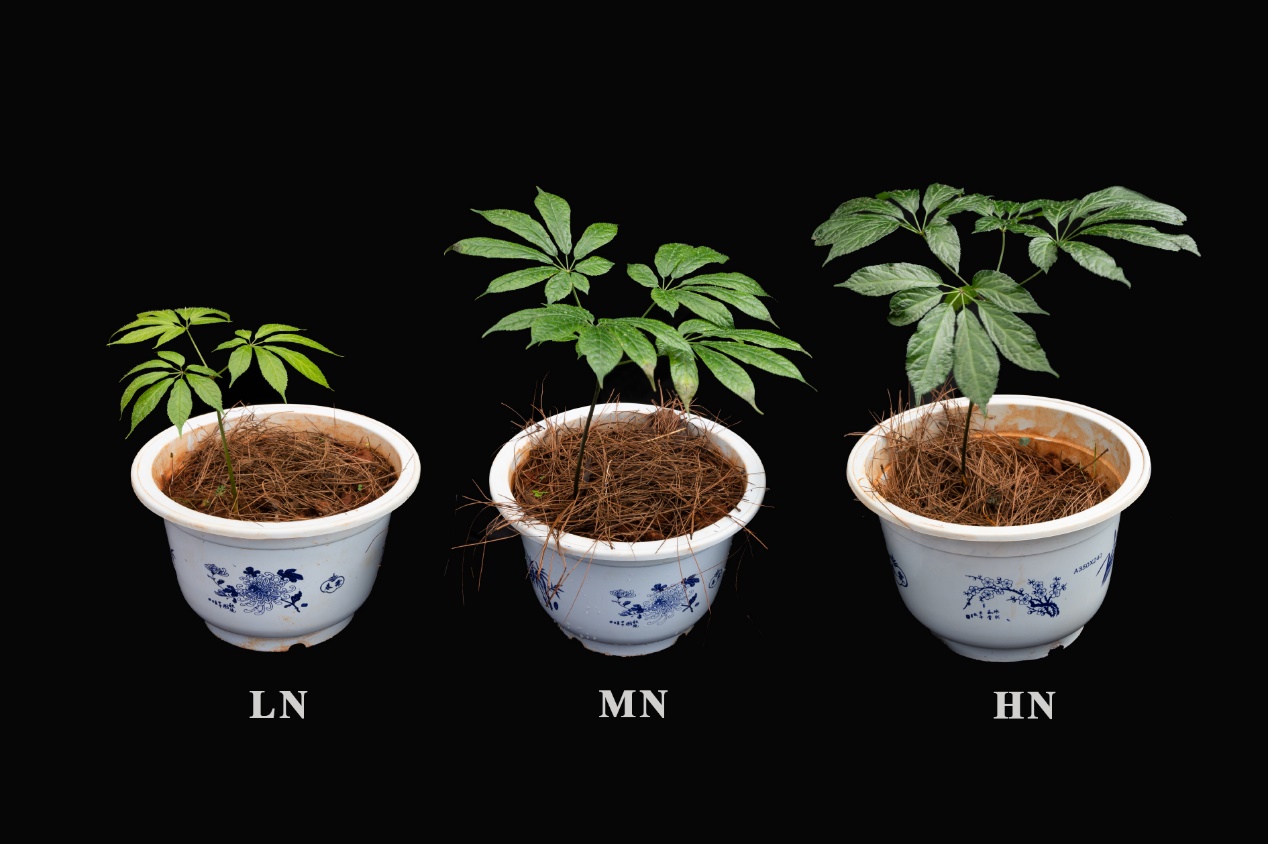


**Figure S3** HPLC chromatograms of *Panax notoginseng* grown under different nitrogen levels.


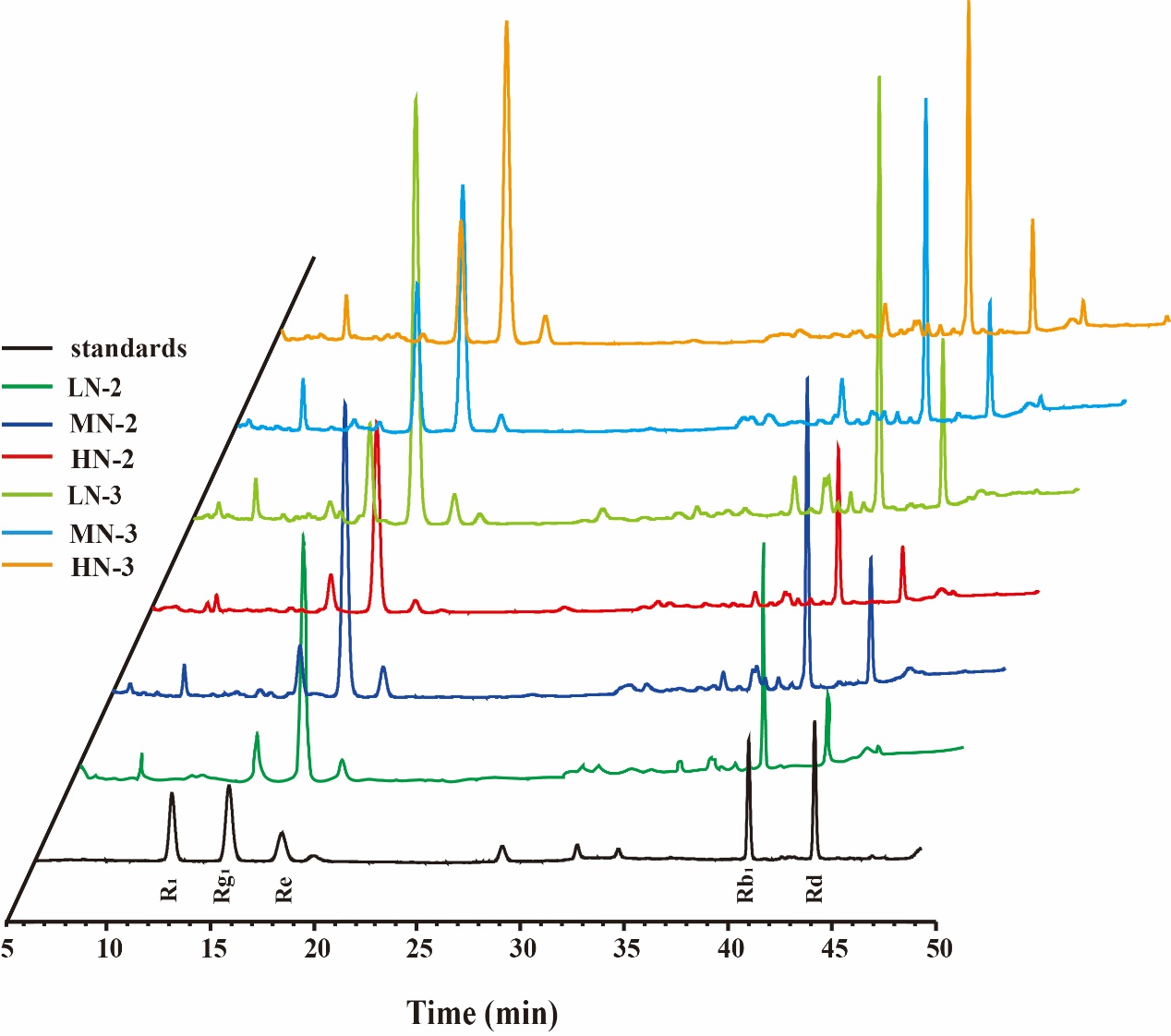

Supplement: Supplemental Information 1 [file peerj-11-14933-s001.docx]
