# Supplementary material for: High nitrogen inhibits biomass and saponins accumulation in a medicinal plant Panax notoginseng
Source: PeerJ. 2023 Feb 21;11:e14933. doi: 10.7717/peerj.14933 (PMC9951802; doi:10.7717/peerj.14933)
Supplement: Supplemental Information 2 [file peerj-11-14933-s002.docx]

**Table S1 Factor loadings and the explained percentage of variance of the first three principal components**

| Variable | Component 1 | Component 2 | Component 3 |
| --- | --- | --- | --- |
| *P*_n_ | -0.14894 | 0.02038 | 0.5247 |
| SLN | -0.28662 | 0.19083 | 0.03506 |
| SPAD | -0.3269 | -0.0657 | -6.20E-05 |
| Chl | -0.12077 | 0.37076 | -0.006 |
| Chl *a* | -0.13741 | 0.32083 | 0.09591 |
| Chl *b* | -0.06417 | 0.33713 | -0.14575 |
| N(root) | 0.16563 | 0.33936 | 0.05863 |
| m(root) | 0.28122 | -0.07693 | 0.26742 |
| N(stem) | -0.31809 | 0.01394 | 0.02845 |
| m(stem) | 0.26117 | 0.24266 | -0.01101 |
| N(leaf) | -0.21127 | 0.26089 | -0.01255 |
| m(leaf) | 0.26814 | 0.23457 | 0.00394 |
| Saponins | 0.19773 | -0.12398 | 0.18203 |
| Height | 0.21885 | 0.26396 | 0.07452 |
| S(leaf) | 0.13654 | 0.35088 | 0.05397 |
| PNUE | -0.22435 | -0.21691 | 0.29524 |
| Yield | 0.28122 | -0.07693 | 0.26742 |
| NUE | 0.29408 | -0.11509 | 0.20104 |
| LCP | 0.16972 | -0.09884 | -0.43008 |
| LSP | 0.09188 | -0.13194 | -0.43734 |
| Percentage of Variance (%) | 44.5% | 28.6% | 11.7% |
| Cumulative % | 44.5% | 28.6% | 84.8% |

*P*_n_: Net photosynthetic rate under saturated light; SLN: Nitrogen content per unit leaf area; Chl: Chlorophyii; Chl *a*: Chlorophyll *a*; Chl *b*: Chlorophyll *b*; N(root): N content in root; m(root): Root biomass; N(stem): N content in stem; m(stem): Stem biomass; N(leaf): N content in leaf; m(leaf): Leaf biomass; S(leaf): Leaf area; PNUE: Photosynthetic N use efficiency; NUE: N use efficiency; LCP: Light compensation point; LSP: Light saturation point
