# Supplementary figures and images for: High nitrogen inhibits biomass and saponins accumulation in a medicinal plant Panax notoginseng
Source: PeerJ. 2023 Feb 21;11:e14933. doi: 10.7717/peerj.14933 (PMC9951802; doi:10.7717/peerj.14933)

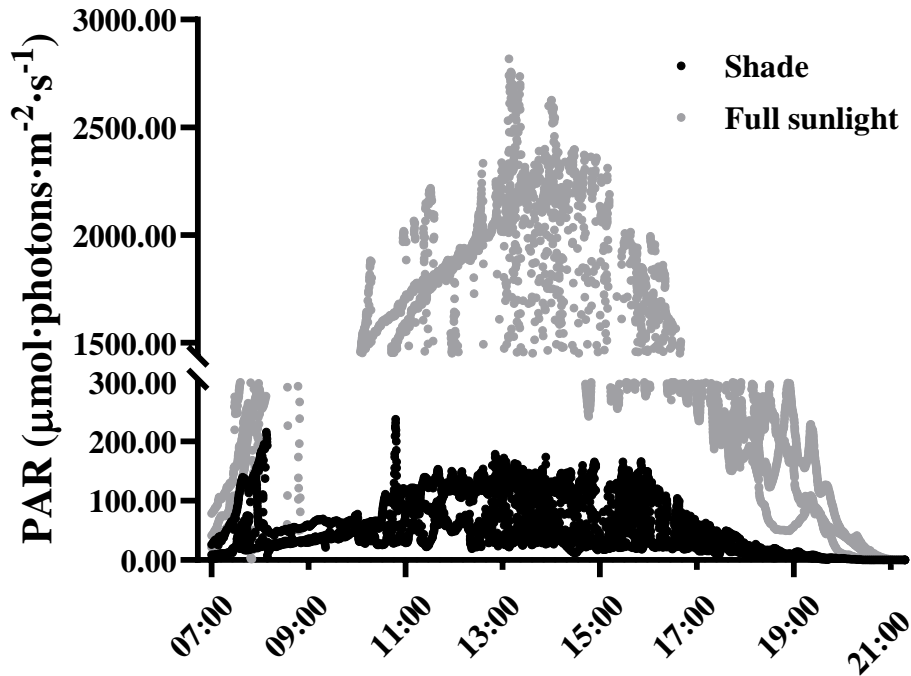

Supplement: Supplemental Information 3 [file peerj-11-14933-s003.zip › Raw Data-20230112/Figure S1.pdf]

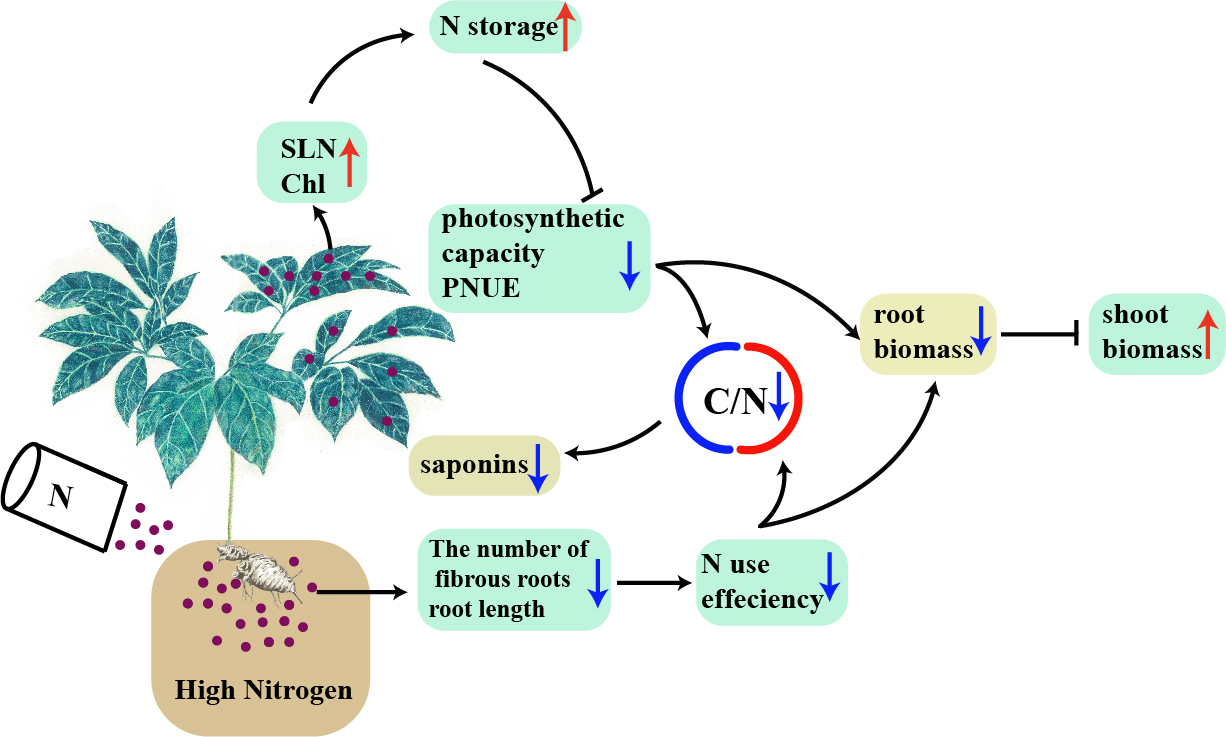

Supplement: Supplemental Information 3 [file peerj-11-14933-s003.zip › Raw Data-20230112/Figure-10-20230112.jpg]

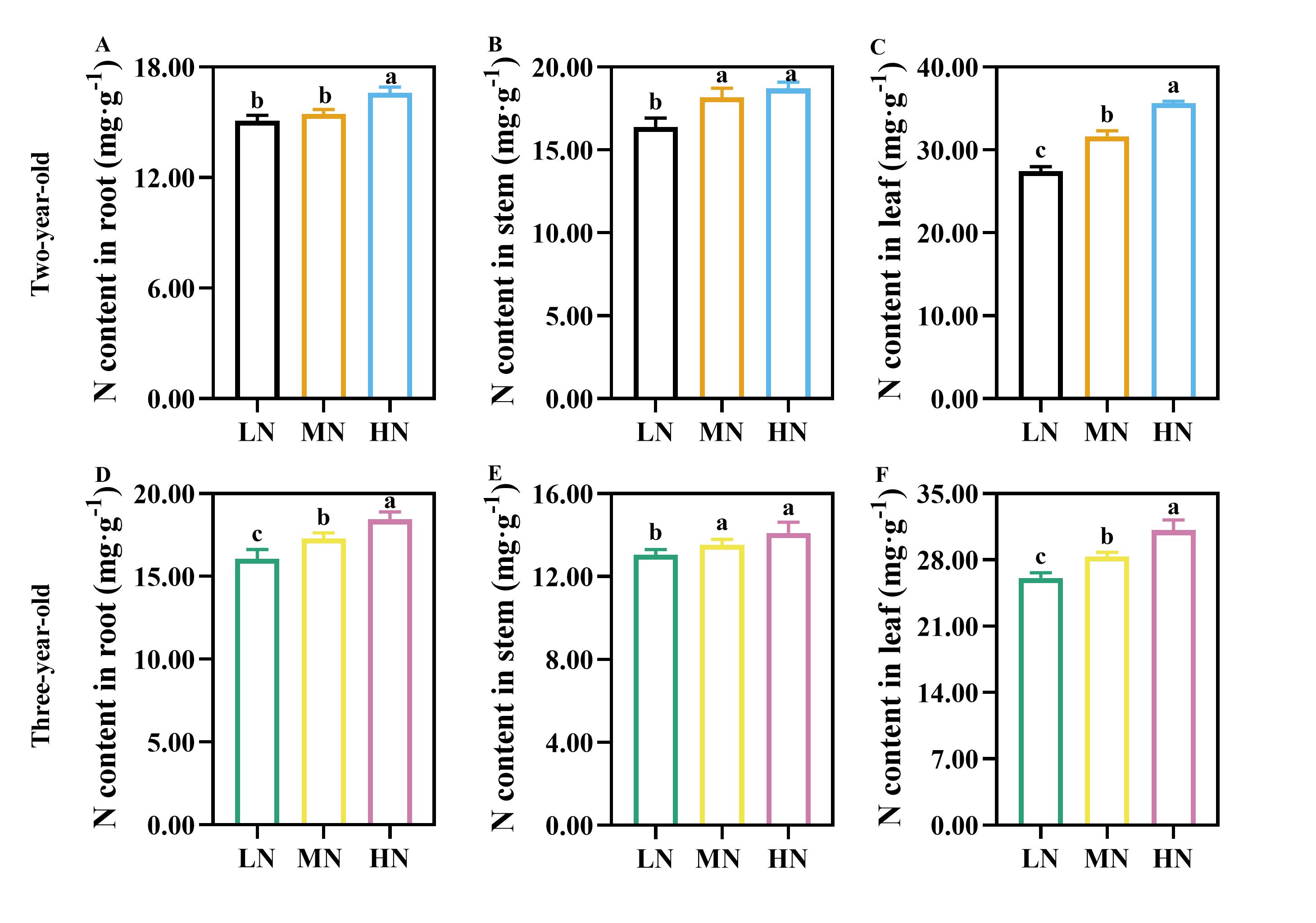

Supplement: Supplemental Information 3 [file peerj-11-14933-s003.zip › Raw Data-20230112/Figure-1-20230112.jpg]

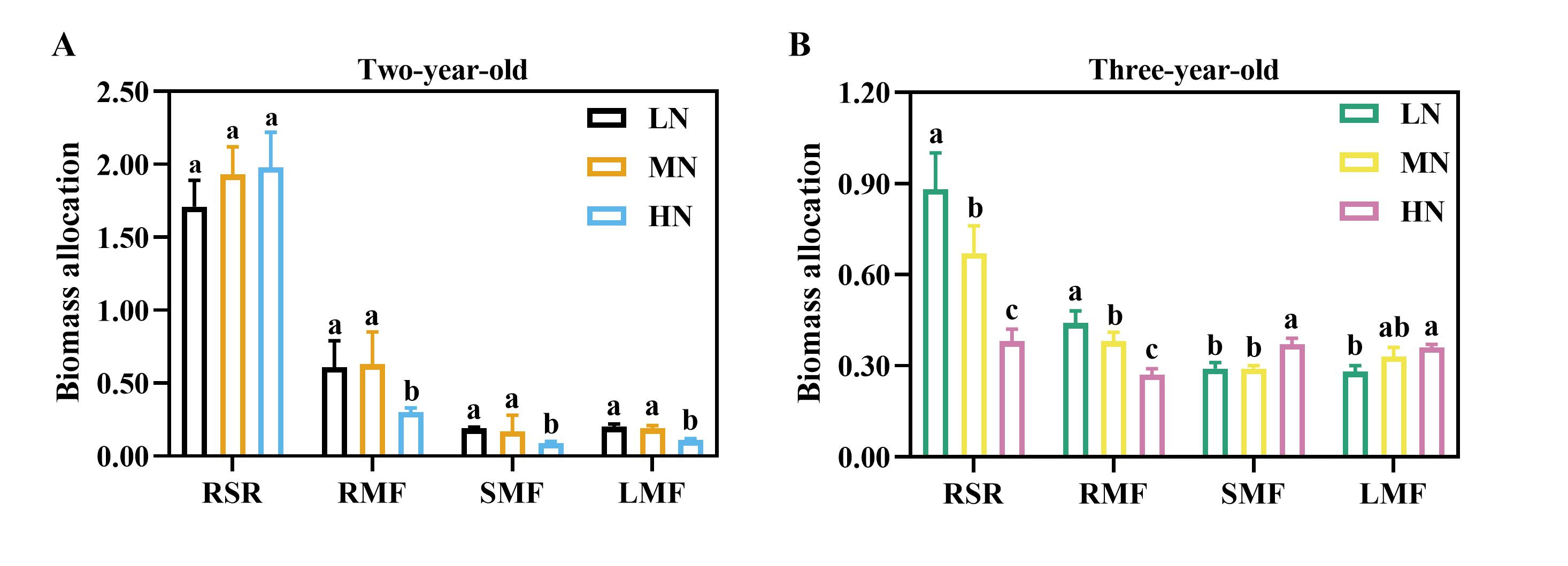

Supplement: Supplemental Information 3 [file peerj-11-14933-s003.zip › Raw Data-20230112/Figure-2-20230112.jpg]

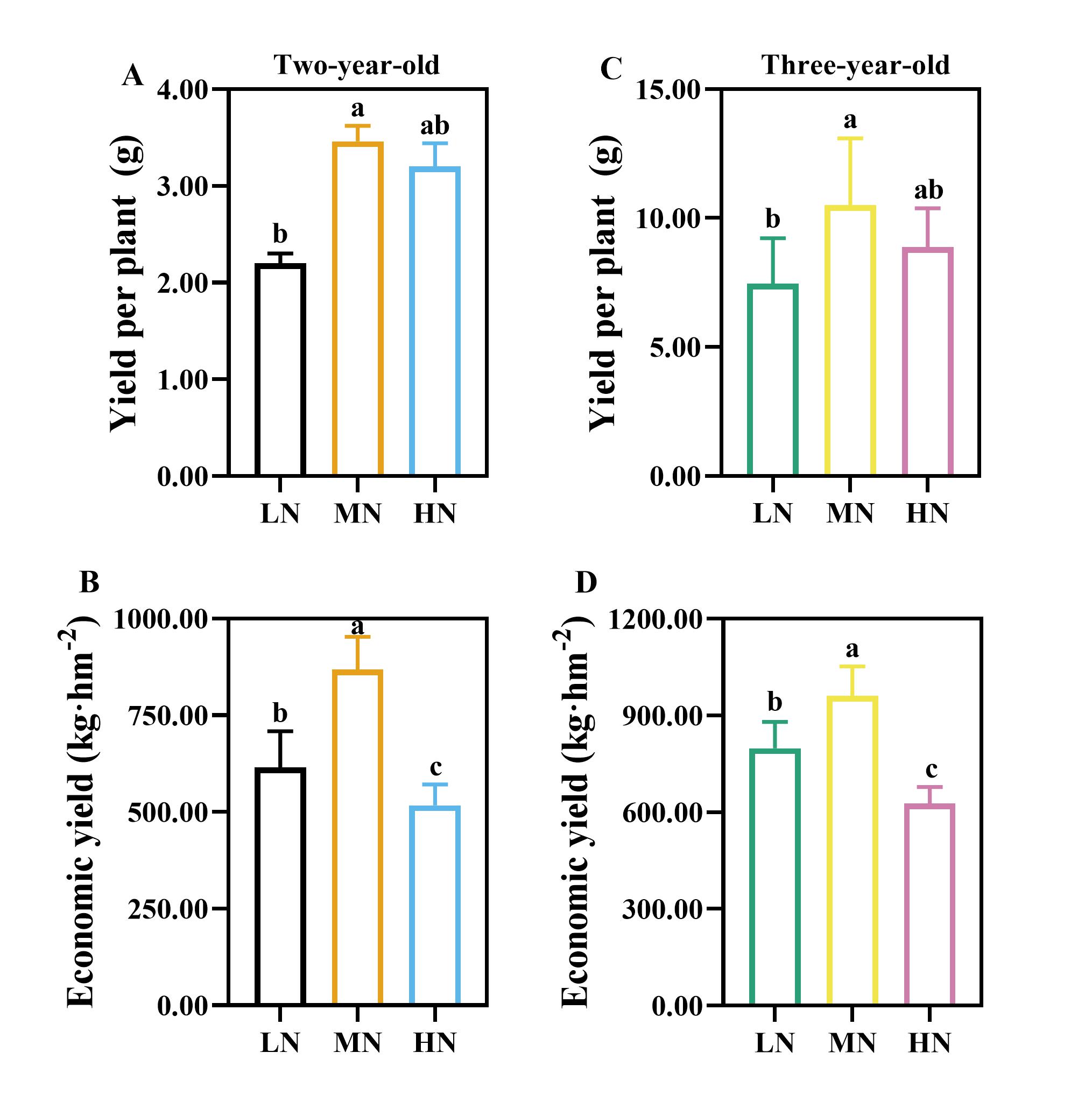

Supplement: Supplemental Information 3 [file peerj-11-14933-s003.zip › Raw Data-20230112/Figure-3-20230112.jpg]

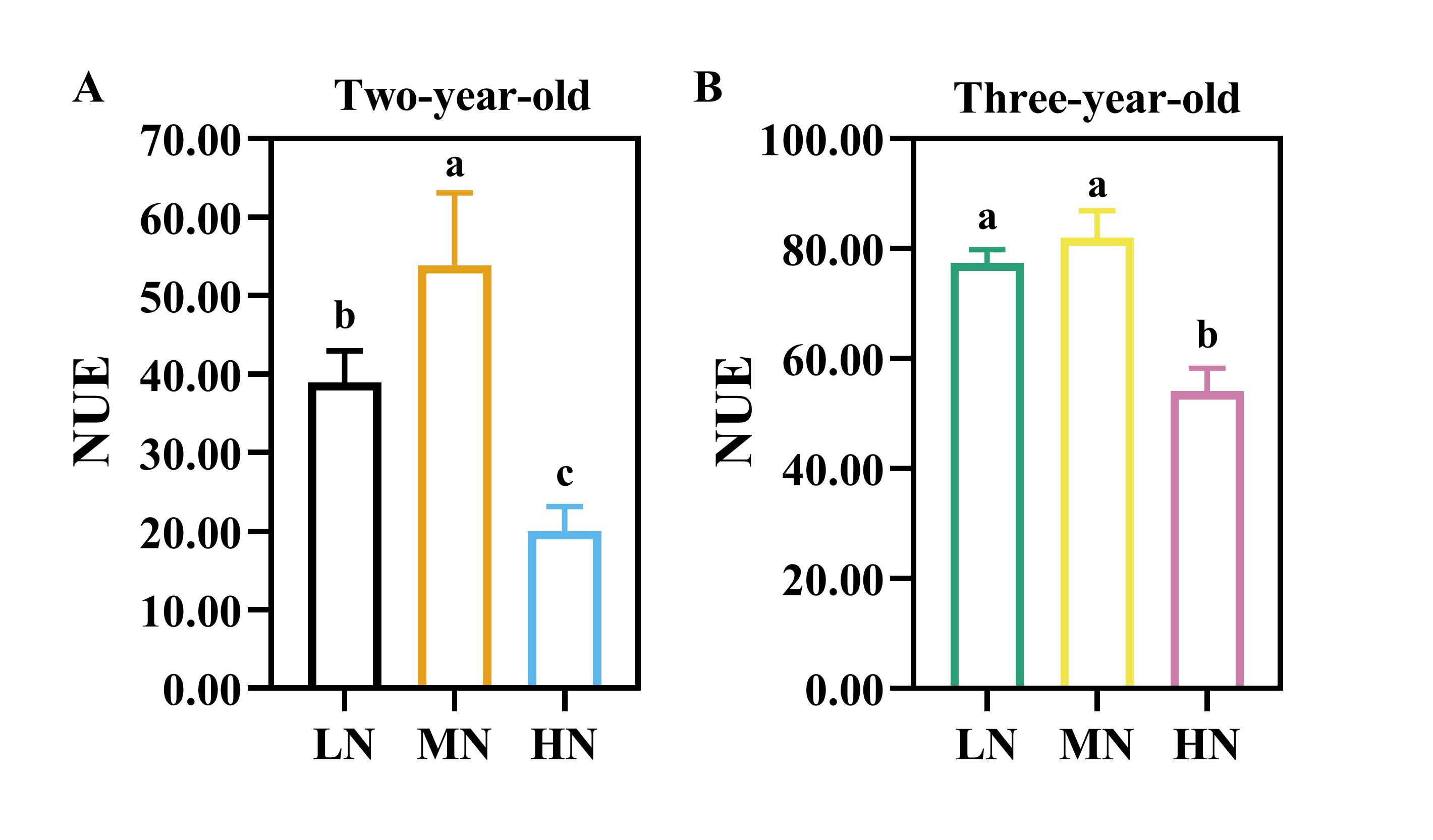

Supplement: Supplemental Information 3 [file peerj-11-14933-s003.zip › Raw Data-20230112/Figure-4-20230112.jpg]

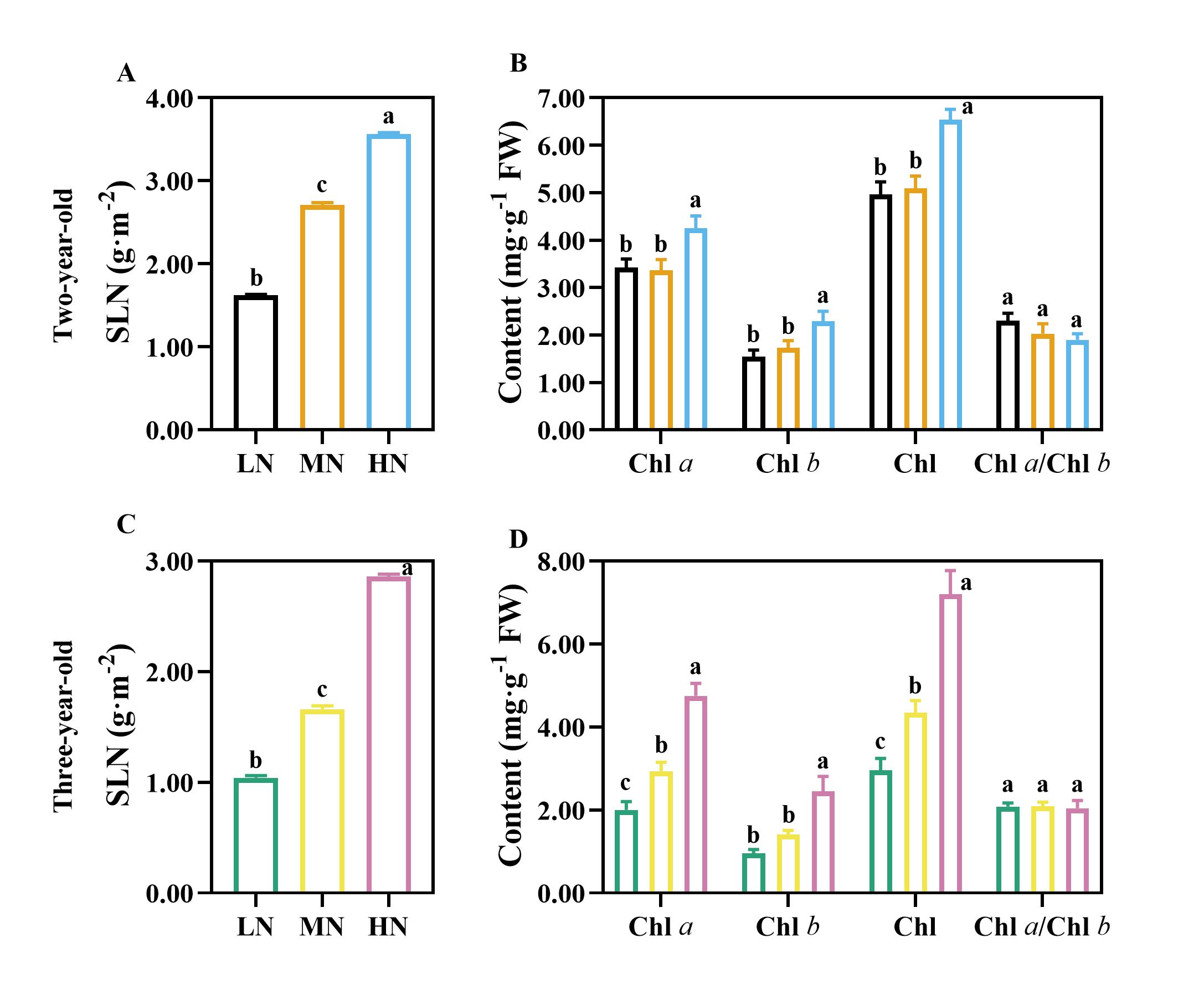

Supplement: Supplemental Information 3 [file peerj-11-14933-s003.zip › Raw Data-20230112/Figure-5-20230112.jpg]

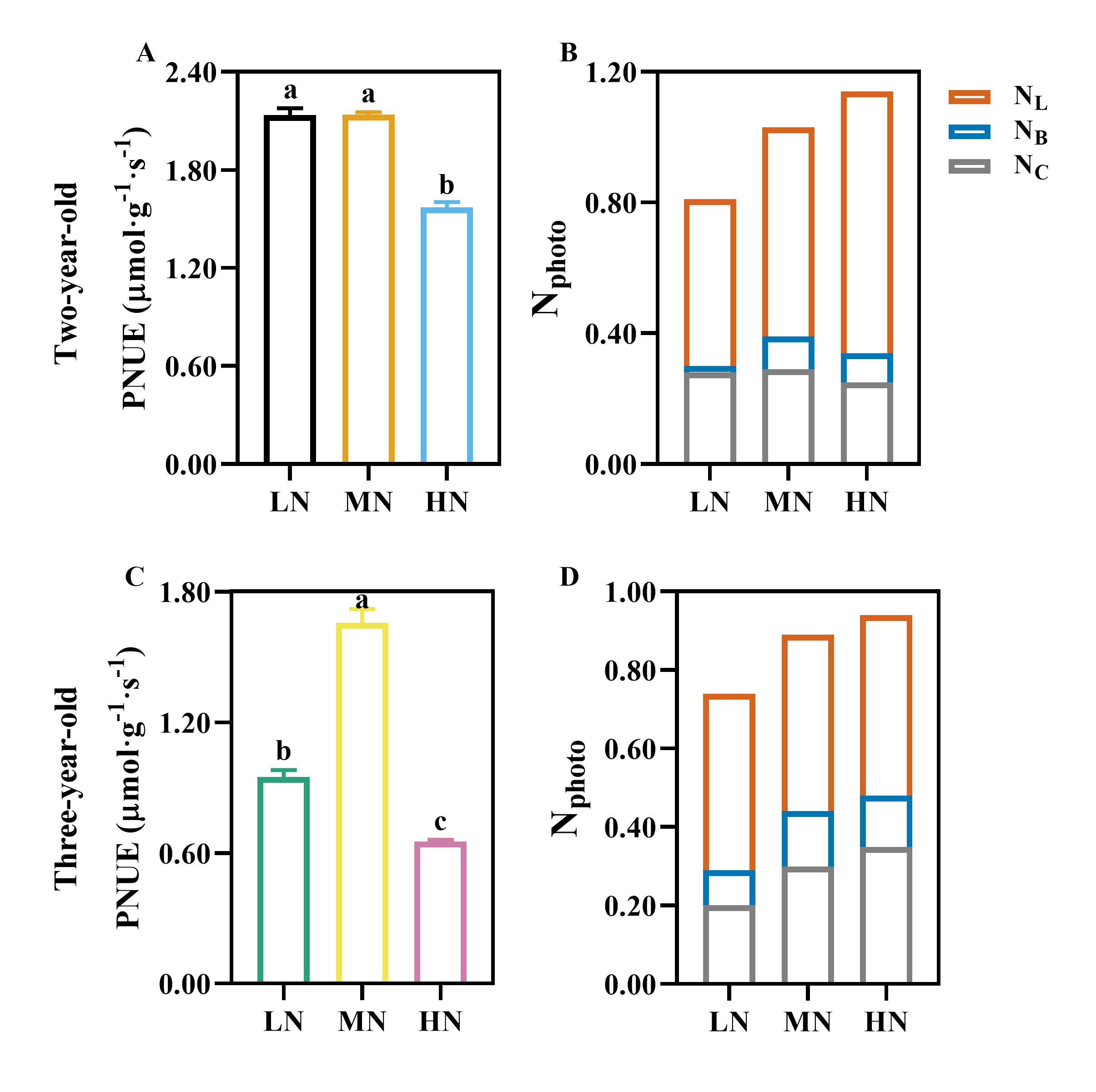

Supplement: Supplemental Information 3 [file peerj-11-14933-s003.zip › Raw Data-20230112/Figure-6-20230112.jpg]

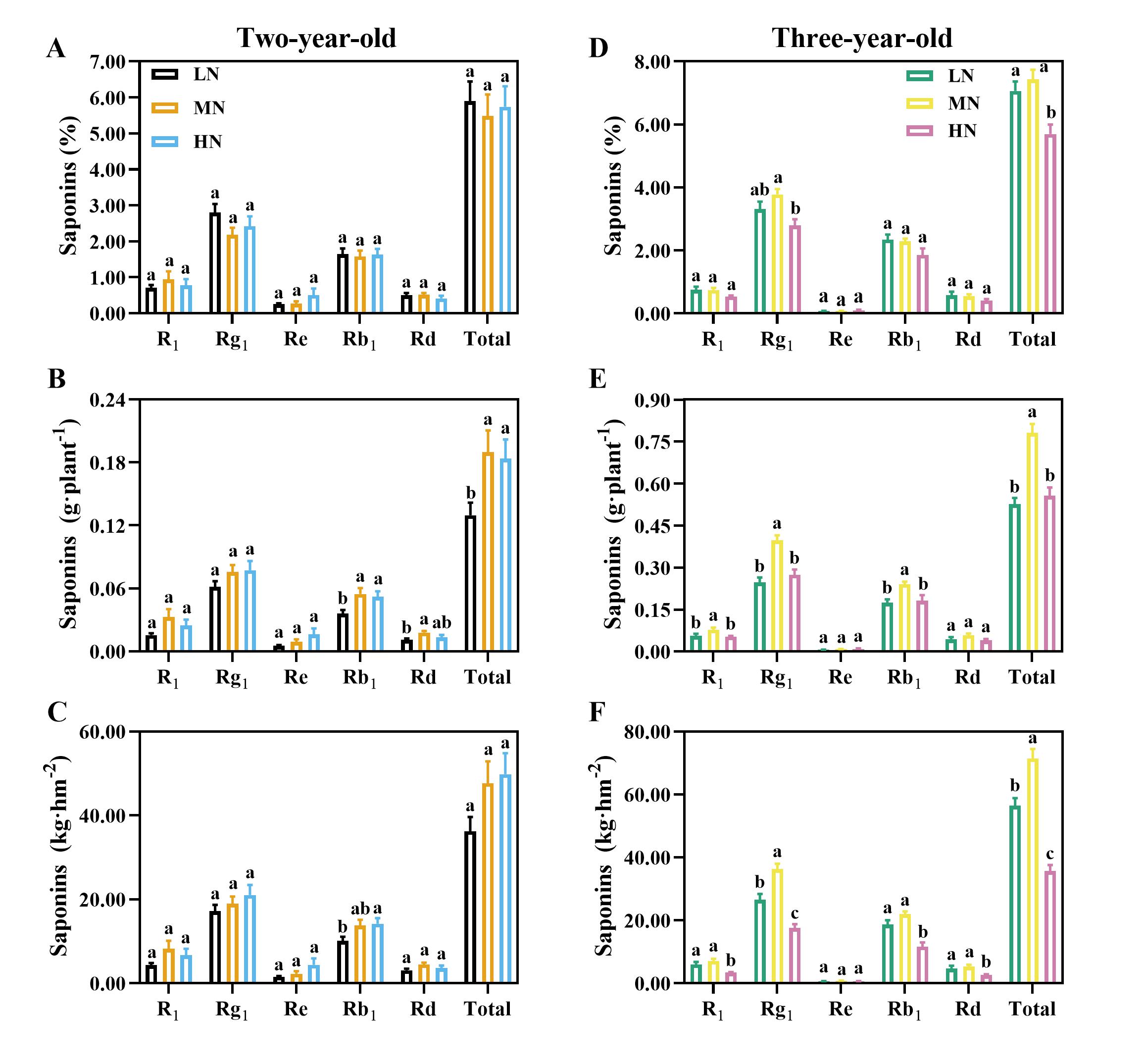

Supplement: Supplemental Information 3 [file peerj-11-14933-s003.zip › Raw Data-20230112/Figure-7-20230112.jpg]

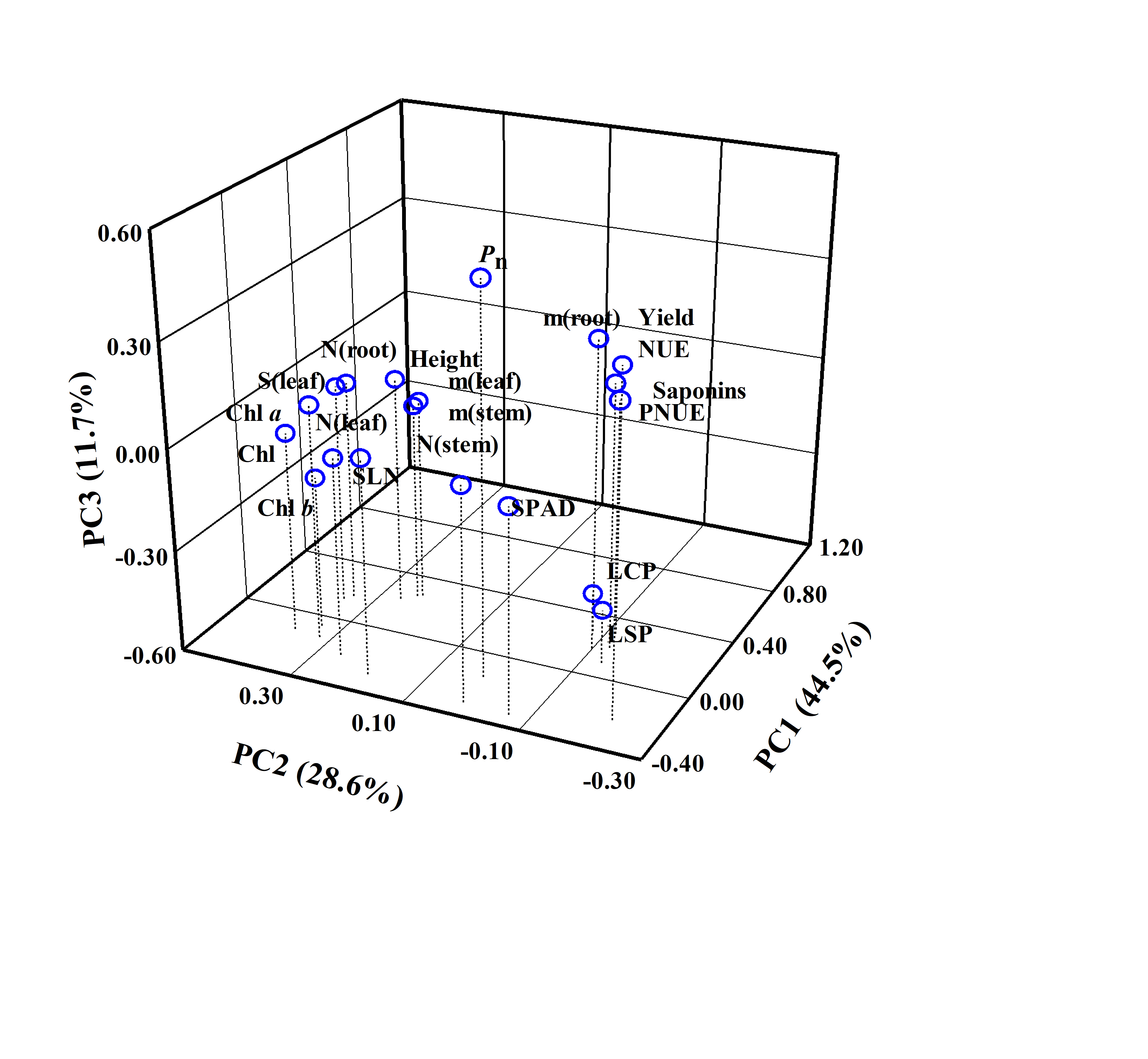

Supplement: Supplemental Information 3 [file peerj-11-14933-s003.zip › Raw Data-20230112/Figure-9-20230112.JPG]
